# Emotional Modulation of the Attentional Blink Is Awareness-Dependent

**DOI:** 10.1371/journal.pone.0046394

**Published:** 2012-09-27

**Authors:** Wenli Qian, Qianli Meng, Lin Chen, Ke Zhou

**Affiliations:** 1 State Key Laboratory of Brain and Cognitive Science, Institute of Biophysics, Chinese Academy of Sciences, Beijing, People's Republic of China; 2 Graduate University of Chinese Academy of Sciences, Beijing, People's Republic of China; 3 Laboratory of Primate Cognitive Neuroscience, Kunming Institute of Zoology, Chinese Academy of Sciences, Kunming, Yunnan, People's Republic of China; University of Florida, United States of America

## Abstract

It is well known that emotion can modulate attentional processes. Previous studies have shown that even under restricted awareness, emotional facial expressions (especially threat-related) can guide the direction of spatial attention. However, it remains unclear whether emotional facial expressions under restricted awareness can affect temporal attention. To address this issue, we used a modified attentional blink (AB) paradigm in which masked (Experiment 1) or unmasked (Experiment 2) emotional faces (fearful or neutral) were presented before the AB sequence. We found that, in comparison with neutral faces, masked fearful faces significantly decreased the AB magnitude (Experiment 1), whereas unmasked fearful faces significantly increased the AB magnitude (Experiment 2). These results indicate that effects of emotional expression on the AB are modulated by the level of awareness.

## Introduction

Emotional facial expressions are considered important for emotional response and social communication, and are particularly important in guiding human motivation [1]. It has been suggested that the human brain may have a specific neural system for efficient and relatively automatic detection of emotional information for faces, especially threat-related information (e.g., fear or anger) [2]–[5]. Recent neuroimaging studies using a backward-masking method to restrict awareness of emotional faces have shown that facial emotions (especially fear-related stimuli) could still activate the corresponding neural structures (e.g., amygdala) even with a lack of explicit knowledge about the presentation of emotional faces stimuli [6]–[9].

Given the special status of the processing of emotional facial expressions, a larger number of studies have investigated the interaction between emotion and attention. These studies revealed a significant emotional modulation of attentional processing. For example, studies on the emotional modulation of spatial attention have shown that spatial cueing by emotional stimuli can guide the direction of spatial attention [4], [10]–[16]. This emotional modulation of spatial attention exists even when threat-related faces are presented under restricted awareness. For instance, in a dot-probe task, Mogg and Bradley (1999) found that angry faces presented under restricted awareness could guide preferential attention to their location [10].

Similarly, the interaction between emotion and temporal attention has been investigated by using the attentional blink (AB) paradigm [17]–[25]. AB refers to a robust psychological phenomenon in which, when viewing a rapid serial visual presentation (RSVP), the detection of an initial target (T1) leads to reduced ability to report subsequent targets (T2) within a brief period (200–500 ms) [26]–[29]. AB has become one of the most widely employed procedures for understanding the temporal limits of attention, and various theoretical frameworks have been proposed to explain the mechanism underlying the AB [30], [31]. Recent research has demonstrated that leading emotional stimuli could affect subsequent cognitive processing [21]–[25], [32], [33]. It has been reported that emotional stimuli that were not to be reported captured temporal attention and led to a deterioration of subsequent target performance with short lags [21]–[25]. For instance, high-arousing pictures interfered with target report [24], [25]. Similar effects have been found when using emotionally arousing words instead of pictures [21], [22]. Moreover, some research has shown that when T1 were fearful or sad faces that were to be reported, the AB could be deteriorated [32], [33].

Furthermore, recent studies have revealed that the emotional modulation of attentional state affects the magnitude of the AB [34]–[37]. Positive stimuli presented before the AB sequence can largely reduce the size of the AB. Using a similar method, fearful faces have been found to increase the AB effect compared with disgust faces [35], [37]. However, given that the emotional stimuli (e.g., faces or scenes) were always visible (e.g., 250 or 1000 ms presentation) in these studies, it remains unclear whether emotional facial expressions presented under restricted awareness, especially threat-related stimuli (i.e., fearful faces), affect temporal attention, as found in the studies of emotional modulation of spatial attention [10], [12].

In the present study, we thus investigated whether the emotional modulation of temporal attention is awareness dependent. We combined an AB paradigm and a masking approach to assess emotional modulation of temporal attention at different awareness levels, in which a masked emotional face appeared before the RSVP sequence. A separate objective forced-choice experiment was carried out to ensure that the awareness level of emotional faces could be successfully manipulated by current masking parameters (e.g., the duration of the target and mask stimuli) used for main experiments (see Supporting Information S1 for details). These parameters were then adopted to examine the influence of fear emotions under restricted awareness on the AB magnitude in Experiment 1 and the effect of conscious fear emotions on the AB magnitude in Experiment 2, respectively.

## Materials and Methods

### Subjects

Forty-two right-handed undergraduate students participated in this study for pay (Experiment 1: 12 females, 9 males, mean age 22; Experiment 2: 11 females, 10 males, mean age 22). All participants had normal or corrected-to-normal visual acuity. All participants provided written informed consent for the study, which was performed according to the principles expressed in the Declaration of Helsinki, and approved by the Human Research Ethics Committee of the Institute of Biophysics, Chinese Academy of Sciences.

### Stimuli

The emotional faces were color photographs which were selected from the Japanese and Caucasian Facial Expressions of Emotion (JACFEE) and Neutral Faces (JACNeuF) set by Matsumoto and Ekman (1988) [38]. These emotional facial expressions included 8 fearful and 24 neutral faces. Each category of faces was balanced by gender. The facial stimuli subtended approximately 7.8° of the visual angle in height and 7.4° of the visual angle in width.

The stimuli in the RSVP included 8 single digits and 24 capital letters, and subtended approximately 0.67×0.95°. In this study, *0*, *1*, *O*, and *I* were omitted to avoid confusion. In each stream, 16 items (14 digits and 2 letters) were included. The digits on a given trial were randomly generated by the computer, and two randomly different uppercase letters were assigned as Tl and T2. T1 could appear in stream positions 3, 4, 5, 6, or 7 (randomly determined), and the temporal distance between T1 and T2 was systematically varied from 1 to 8 items (Lags 1–8, respectively). All stimuli were draw in black on a gray (25 cd/m^2^) background.

### Procedures

All stimuli were presented on a 19-in CRT monitor with a resolution of 1024×768 pixels and a refresh rate of 100 Hz. The stimuli were viewed at a distance of 90 cm. Each subject was tested individually in a dimly lit room. The subjects began each trial by pressing the space bar on the computer keyboard. Ten to twenty practice trials were conducted before each experiment.

**Figure 1:**
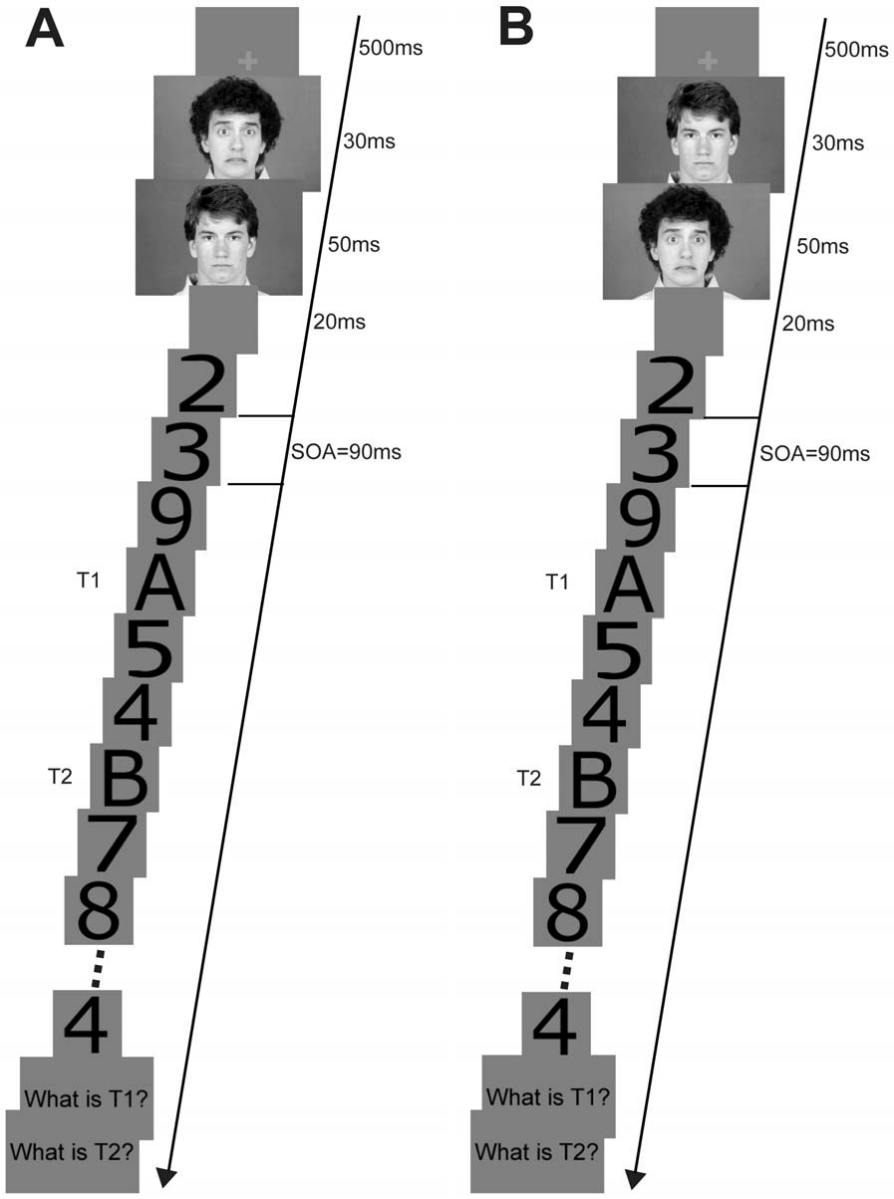
Schematic illustration of the procedures. (A) Experiment 1; (B) Experiment 2.

Experiment 1 consisted of 320 trials. As shown in Figure 1A, each trial began with a 500-ms presentation of a green fixation cross (0.11×0.11°) at the center of the screen. Subjects were asked to fixate on the central cross. After the fixation cross went off, an emotional face (neutral or fearful) appeared for 30 ms and was immediately backward masked with a neutral face lasting 50 ms. In previous studies, a 30-ms presentation of a target-mask SOA has been regarded as sufficiently brief to prevent awareness of facial expressions in previous studies [6], [14], [39], [40; see also Supporting Information S1]. Upon offset of the masked emotional face and a 20-ms gap, the stream of stimuli in the RSVP appeared successively without interstimulus blanks at the center of the screen. Each stimulus was presented for 90 ms. When the task introduction appeared on the screen, the subjects were instructed to report the identity of the two letters in presentation order by pressing the corresponding keys on the keyboard. Subjects were encouraged to avoid making wild guesses and no feedback was given.

The stimuli and procedure in Experiment 2 were similar to those in Experiment 1, except that the presentation order between emotional faces (fearful or neutral faces) and backward masks (neutral faces) was transposed. That is, a 30-ms neutral face was masked by a 50-ms fearful or neutral face (Figure 1B).

## Results

### Experiment 1: masked fearful faces decreased the AB effects

**Figure 2:**
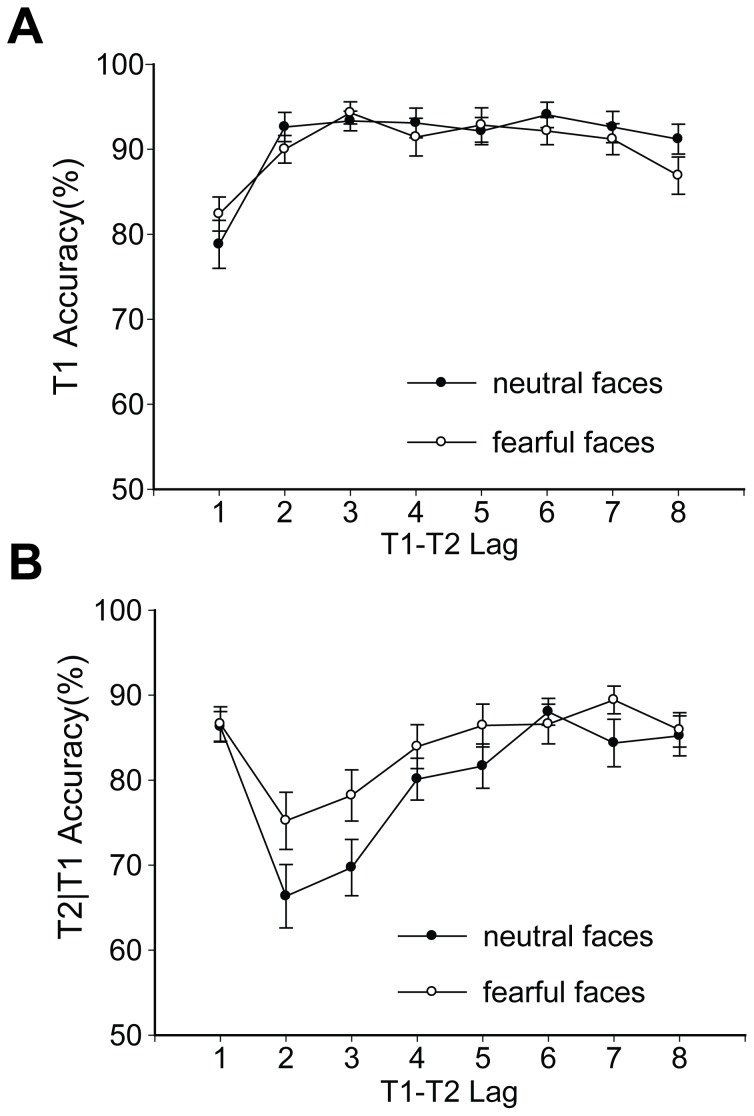
The average identification accuracy of targets in different emotional conditions of Experiment 1. (A) T1 performance; (B) T2 performance. The results are shown separately for the neutral and fearful conditions. Error bars represent one standard error of the mean.

A repeated measures ANOVA was employed to analyze the mean T1 and T2 accuracy with the emotional condition (neutral and fearful) and T2 lag (1–8) as variables. Trials in which T1 and T2 were correctly identified, regardless of the report order, were treated as correct. The results for T1 are shown in Figure 2A. There was a significant main effect of T2 lag (*F*_7, 140_ = 14.771, *p*<0.001, *η*_p_^2^ = 0.425). T1 performance was quite poor for Lag 1, similar to previous findings [41], [42], [43]. The close temporal proximity of T1 and T2 may lead to competition for attention; thus, better T2 performance (lag-1 sparing) may cause worse T1 performance [41], [42]. Moreover, masked emotional faces did not affect T1 accuracy. There were no significant interaction between emotional condition and T2 lag (*F*_7, 140_ = 1.406, *p* = 0.207, *η*_p_^2^ = 0.066) and no significant main effect of emotional condition (*F*_1, 20_ = 1.286, *p* = 0.270, *η*_p_^2^ = 0.06).

Figure 2B illustrates T2 performance based on the trials for the correct identification of T1. The data showed a significant main effect of T2 lag (*F*_7, 140_ = 15.015, *p*<0.001, *η*_p_^2^ = 0.429), a reliable main effect of emotional condition (*F*_1, 20_ = 14.988, *p* = 0.001, *η*_p_^2^ = 0.428) and a significant interaction between emotional condition and T2 lag (*F*_7, 140_ = 2.282, *p* = 0.031, *η*_p_^2^ = 0.102). AB effects were obtained in both emotional conditions in which T2 accuracy dropped after lag 1 and then gradually increased again (all *F*s>5, *p*s<0.001, *η*_p_^2^>0.2). Then, Bonferroni-corrected t tests were used to investigate differences in T2 performance at each lag between fearful and neutral conditions. 0.05 was chosen as the significant level and divided it by the eight pairwise comparisons. A significant level of 0.0063 was yielded (*α* = 0.0063). The data revealed that T2 accuracy was significantly higher in the fearful condition than in the neutral condition only at Lag 2 (*t*_20_ = 4.018, *p* = 0.001, Cohen's *d* = 0.877), which was similar to the finding of previous study [32]. In the majority of AB studies, the AB is accentuated at lag 2 since items closely succeeding T1 were forced to compete for attentional resources already engaged by T1 [28], [44]. Thus, the present results showed that the masked fearful faces could attenuate the AB deficit in comparison with the masked neutral faces.

### Experiment 2: unmasked fearful faces increased the AB effects

**Figure 3:**
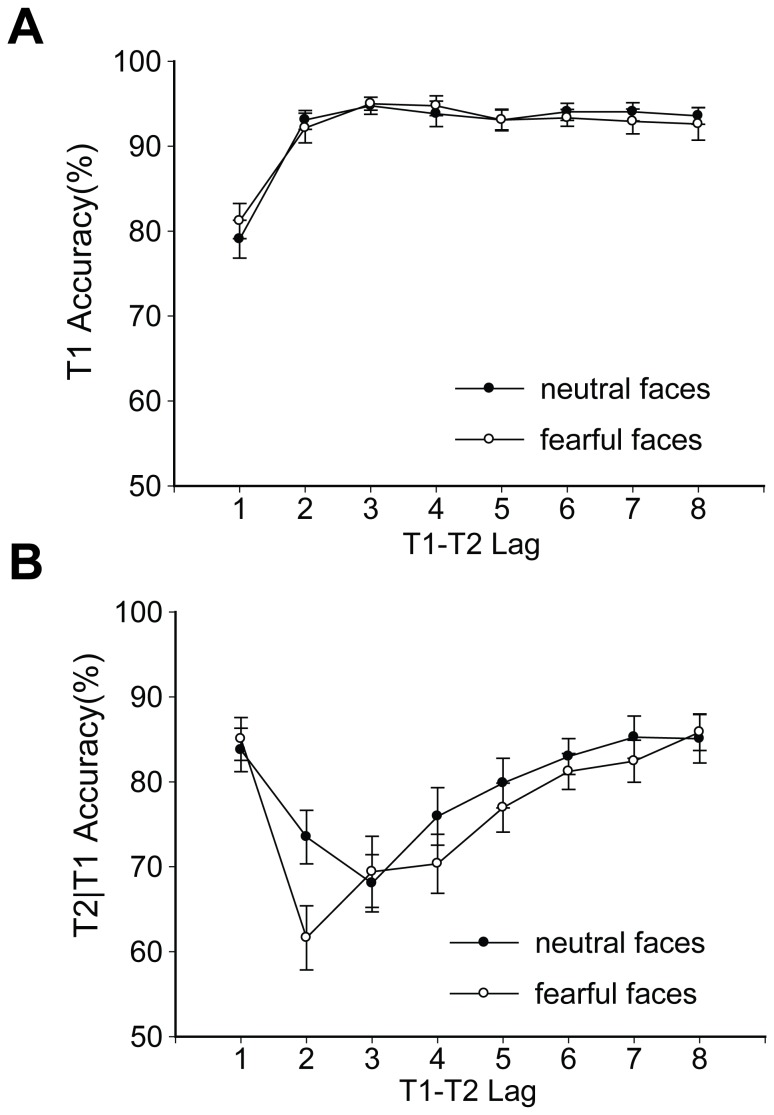
The average identification accuracy of targets in different emotional conditions of Experiment 2. (A) T1 performance; (B) T2 performance. The results are shown separately for the neutral and fearful conditions. Error bars represent one standard error of the mean.

The analysis method used in Experiment 2 was exactly the same as that used in Experiment 1. As shown in Figure 3A, consistent with the findings in Experiment 1, unmasked emotional faces did not affect T1 performance. Only a significant main effect of T2 lag (*F*_7,__140_ = 24.431, *p*<0.001, *η*_p_^2^ = 0.550) was found, with the lowest accuracy at Lag 1. There were no other significant effects (main effect of emotional condition, *F*_1, 20_ = 0.013, *p* = 0.909, *η*_p_^2^ = 0.001; interactive effect of emotional condition and T2 lag, *F*_7, 140_ = 0.536, *p* = 0.806, *η*_p_^2^ = 0.026).

For T2 performance, as illustrated in Figure 3B, the data revealed a significant main effect of T2 lag (*F*_7, 140_ = 12.218, *p*<0.001, *η*_p_^2^ = 0.379) and a significant emotional condition×T2 lag interaction (*F*_7, 140_ = 2.258, *p* = 0.033, *η*_p_^2^ = 0.101). Obvious AB effects were found for both emotional conditions (all *F*s>6, *p*s<0.001, *η*_p_^2^>0.2). Significantly, we found that the effect of emotional facial expressions on the AB was very different from that found in Experiment 1. T2 accuracy was significantly lower in the fearful condition than in the neutral condition (main effect of emotional condition, *F*_1, 20_ = 12.566, *p* = 0.002, *η*_p_^2^ = 0.386). The planned t tests with Bonferroni-correction revealed that there was a significant difference between the fearful and neutral conditions at Lag 2 (*t*_20_ = 3.527, *p* = 0.002, Cohen's *d* = 0.770).

To further confirm whether different awareness levels of fearful faces have distinct emotional modulation of the AB magnitude. A three-way ANOVA was used to analyze the mean T2 accuracies between two experiments with the awareness level (restricted awareness in Experiment 1 and conscious awareness in Experiment 2), emotional condition (neutral and fearful), and T2 lag (1–8) as variables. The analysis revealed a significant interaction between emotional condition and awareness level (*F*_1, 40_ = 27.347, *p*<0.001, *η*_p_^2^ = 0.406), a significant interaction between emotional condition, T2 lag and awareness level (*F*_7, 280_ = 3.369, *p* = 0.002, *η*_p_^2^ = 0.078), a significant main effect of T2 lag (*F*_7, 280_ = 25.551, *p*<0.001, *η*_p_^2^ = 0.390) and a marginally significant main effect of awareness level (*F*_1, 40_ = 3.064, *p* = 0.088, *η*_p_^2^ = 0.071). There were no other significant effects (all *F*s<1.2, *p*s>0.3, *η*_p_^2^<0.03). T2 performance was significantly higher in the masked fear condition than in the unmasked fear condition (main effect of awareness level, *F*_1, 40_ = 9.452, *p* = 0.004, *η*_p_^2^ = 0.191; interaction between T2 lag and awareness level, *F*_7, 280_ = 2.283, *p* = 0.028, *η*_p_^2^ = 0.054). However, there was no significant difference in the neutral condition between two experiments (main effect of awareness level, *F*_1, 40_ = 0.134, *p* = 0.716, *η*_p_^2^ = 0.003; interaction between T2 lag and awareness level, *F*_7, 280_ = 1.385, *p* = 0.211, *η*_p_^2^ = 0.033). Thus, compared with neutral faces, fearful faces with restricted awareness may attenuate the AB effect, while briefly presented fearful faces with awareness may deteriorate the AB deficit.

## Discussion

It is well known that emotion can modulate attentional processes. Previous studies have revealed that emotional faces, especially threat-related facial stimuli, even under restricted awareness, can guide and affect the direction of spatial attention [10], [12]. However, it remains unknown whether emotional faces presented under restricted awareness can affect temporal attention. In the present study, we manipulated the awareness level of emotional facial stimuli (Experiment 1: masked; Experiment 2: unmasked) presented before the RSVP streams, and examined the influences of emotional facial expressions on the AB effect. The following conclusions can be drawn from the present data: (1) In the masked condition, fearful faces with restricted awareness significantly decreased the size of the AB compared with neutral faces (Experiment 1); (2) In the unmasked condition, conscious fearful faces significantly increased the size of the AB compared with neutral faces (Experiment 2).

Recent AB studies have shown that irrelevant mental activity (e.g., listening to music or task-irrelevant visual motion and flicker) reduced the AB deficit [35], [43], [45]–[47]. If cognitive processing draws attention away from the AB task, such as directing attention to a different spatial location [35], [43], [45], the AB deficit will be attenuated. In contrast, if cognitive processing increases attentional control of the AB task, the AB magnitude will be increased [47]. This result is explained as a function of a diffuse state of attention characterized by a more distribution of attentional resources. When more attentional resources are assigned to the RSVP stream, the greater processing of distractors leads to more interference with targets and increases the probability of an AB [34]. According to this hypothesis, some studies have suggested that positive affect leading to more diffuse attention can decrease the AB, whereas negative affect leading to more focused attention can increase the AB [34]–[37]. Our results thus corroborate and complement these findings in AB studies. The result of Experiment 2 was consistent with previous studies, showing that conscious fearful faces increase the AB [37]. This deteriorated AB may be due to a more focused attentional state caused by unmasked fearful faces [34], [36]. Interestingly, a reverse pattern was found in Experiment 1: fearful faces with restricted awareness reduced the AB magnitude. In terms of the supposition of diffused attention, these attenuated AB effects may result from a more distributed attentional state triggered by the processing of the masked fearful face. The different emotional influences on temporal attention are similar to findings on the emotional modulation of spatial attention. Attention can be oriented to threatening stimuli (e.g., fearful or angry) presented under restricted awareness [10], [48], but such type of automatic attentional capture is not evident when the awareness of emotional stimuli is less restricted or unrestricted [10], [49].

Recent research has explored how emotion modulates temporal attention. For instance, Jefferies et al. (2008) examined how the emotion-attention relationship varies in both mood valence (negative vs. positive) and arousal (low vs. high). They found no differences in T1 accuracy, but T2 accuracy was highest for participants with low arousal and negative affect, lowest for those with strong arousal and negative affect, and intermediate for those with positive affect regardless of arousal [34]. Moreover, using to-be-ignored RSVP distractor to act as T1, it has been found that arousal (e.g., sexual words) rather than negative, positive, threatening, or emotionally neutral stimuli, may lead to lower subsequent target accuracy (T2) [21]. Although these results are divergent, it seems that emotionally arousing stimuli may modulate temporal attention. In the present study, the emotional modulation of temporal attention may result from the association between fearful faces and higher arousal ratings compared to neutral faces.

The present study investigated the interaction between emotion and temporal attention, and found that fearful faces under restricted awareness reduced the AB magnitude, whereas briefly presented fearful faces with awareness increased the AB magnitude. The opposite emotional modulation on the AB at different awareness levels may be caused by the different neural mechanisms underlying the conscious and unconscious emotional processing. Previous studies have suggested that emotional faces, even without awareness, could proceed by a ‘fast’ route, such as a subcortical pathway, which bypasses the primary sensory cortices and relies on crude sensory input in the visual domain [7], [13], [50]–[52]. This processing advantage suggests that unconscious emotion may be more fundamental and more likely to attract attention, leading to a diffused attentional state that reduces the AB effect. However, further investigations using functional MRI (fMRI) and event-related potential (ERP) approaches are needed to clarify this issue.

## Supporting Information

Supporting Information S1 A separate objective forced-choice experiment was carried out to ensure that the awareness level of emotional faces could be successfully manipulated by current masking parameters (e.g., the duration of the target and mask stimuli) used for main experiments. The *d′* measures were computed for each individual. The result revealed that subjects' awareness of the fearful faces was prevented (or restricted) by using a 30-ms presentation of a target-mask SOA (masked condition) and was unrestricted when the fearful faces were presented as briefly as 50 ms (unmasked condition).
(DOC)
