# Supplementary material for: Emotional Modulation of the Attentional Blink Is Awareness-Dependent
Source: PLoS One. 2012 Sep 27;7(9):e46394. doi: 10.1371/journal.pone.0046394 (PMC3459896; doi:10.1371/journal.pone.0046394)
Supplement: Supporting Information S1 — A separate objective forced-choice experiment was carried out to ensure that the awareness level of emotional faces could be successfully manipulated by current masking parameters (e.g., the duration of the target and mask stimuli) used for main experiments. The d′ measures were computed for each individual. The result revealed that subjects' awareness of the fearful faces was prevented (or restricted) by using a 30-ms presentation of a target-mask SOA (masked condition) and was unrestricted when the fearful faces were presented as briefly as 50 ms (unmasked condition). (DOC) [file pone.0046394.s001.doc]

**Supporting Information S1**

Since the major goal of the present study is to investigate whether emotional faces presented under restricted awareness affect temporal attention in the AB　paradigm, it is critical to ensure that the awareness of emotional faces indeed are restricted when using backward masking method. For this reason, the masking parameters (e.g., the presentation duration of the target images and masking images) were manipulated in an objective forced-choice experiment. The *d*' measures were computed for each individual. Using the signal detection theory [1], the objective criterion for lack of conscious perception was *d*' = 0.

**Materials and Methods**

Subjects

Eighteen new undergraduate students (10 females, mean age 23, all right-handed) participated in this experiment. All had normal or corrected-to-normal vision.

Stimuli and Procedures

The subjects were exposed to pairs of facial stimuli, one target and one mask. There were two conditions (masked vs. unmasked), each containing one block of 60 trails. The order of two blocks was counterbalanced across subjects. The emotional faces and masking parameters were exactly the same as those used in Experiment 1 and 2. In the masked condition, a target emotional face was presented for 30 ms and immediately followed by a masking neutral face appearing for 50 ms. Half of the target faces were fearful faces and the remaining 30 targets were neutral faces. Subjects made an objective forced-choice decision about the target facial expression (fearful or neutral) via button-press after each target/mask trial. Note that in this condition, we hypothesized that subjects’ awareness of the fearful faces was prevented by backward masking with another neutral face. In the unmasked condition, the stimuli and procedure were the same as those used in the masked condition except that the presentation order of targets (fearful or neutral faces) and masks (neutral faces) was transposed. That is, a 30 ms neutral face was followed by a 50 ms fearful or neutral face. Subjects also made an objective forced-choice decision about the target facial expression (fearful or neutral). Note that in this condition, we hypothesized that subjects could be aware of the emotional facial expressions.

**Results and Discussion**

We used one-sample t test to evaluate the statistical differences between the discrimination performance of emotional faces and the chance level (*d*' = 0). As shown in Table S1, the data revealed that the discrimination performance in the masked condition (mean=0.11, SE=0.065) was not significantly different from the chance level (*t*_17_=1.718, *p*=0.104), indicating that subjects’ awareness of the fearful faces was prevented (or restricted) by using the current set of masking parameters. Moreover, the mean *d*' values in the unmasked condition (mean=3.94, SE=0.14) were significantly higher than the chance level (*t*_17_=27.896, *p*<0.001), suggesting that subjects could be well aware of the emotional faces even when they were presented as briefly as 50 ms.

**Reference**

1. Macmillan N (1986) The psychophysics of subliminal perception. Behav Brain Sci 9: 38–39.

**Table S1 Mean sensitivity scores (*d*') in two conditions**

| Subject | Masked condition | Unmasked condition |
| --- | --- | --- |
| GYQ | 0.12 | 4.26 |
| WGJ | -0.12 | 4.26 |
| CRJ | 0.48 | 4.26 |
| ZWH | 0.12 | 4.26 |
| ZY | 0.36 | 4.26 |
| XQS | 0.12 | 4.26 |
| LX | -0.24 | 3.29 |
| DC | -0.18 | 3.77 |
| LQL | 0.75 | 4.26 |
| WMK | -0.12 | 4.26 |
| FF | 0.06 | 4.26 |
| FYC | 0.12 | 3.63 |
| WNK | 0.12 | 4.26 |
| WF | 0.24 | 2.12 |
| LXH | 0.31 | 4.26 |
| XSN | -0.43 | 2.94 |
| WXT | 0.06 | 4.26 |
| CF | 0.24 | 3.96 |
